# Comprehensive characterization and clinical relevance of the SWI/SNF copy number aberrations across human cancers

**DOI:** 10.1186/s41065-021-00203-y

**Published:** 2021-10-01

**Authors:** Zhiwei Xing, Buhuan Ma, Weiting Sun, Yimin Sun, Caixia Liu

**Affiliations:** 1 The Affiliated Hospital of Inner Mongolia Medical University, Hohhot, 010050 Inner Mongolia China; 2 Inner Mongolia Medical University, Hohhot, 010050 Inner Mongolia China; 3 National Engineering Research Center for Beijing Biochip Technology, Beijing, 102206 China; 4 Department of Biomedical Engineering, Medical Systems Biology Research Center, Tsinghua University School of Medicine, Beijing, 100084 China

**Keywords:** SWI/SNF complex, Copy number alteration, Immune cell infiltration, Genome instability

## Abstract

**Background:**

Alterations in genes encoding chromatin regulatory proteins are prevalent in cancers and may confer oncogenic properties and molecular changes linked to therapy resistance. However, the impact of copy number alterations (CNAs) of the SWItch/Sucrose NonFermentable (SWI/SNF) complex on the oncogenic and immunologic properties has not been systematically explored across human cancer types.

**Methods:**

We comprehensively analyzed the genomic, transcriptomic and clinical data of The Cancer Genome Atlas (TCGA) dataset across 33 solid cancers.

**Results:**

CNAs of the SWI/SNF components were identified in more than 25% of all queried cancers, and tumors harboring SWI/SNF CNAs demonstrated a worse overall survival (OS) than others in several cancer types. Mechanistically, the SCNA events in the SWI/SNF complex are correlated with dysregulated genomic features and oncogenic pathways, including the cell cycle, DNA damage and repair. Notably, the SWI/SNF CNAs were associated with homologous recombination deficiency (HRD) and improved clinical outcomes of platinum-treated ovarian cancer. Furthermore, we observed distinct immune infiltrating patterns and immunophenotypes associated with SWI/SNF CNAs in different cancer types.

**Conclusion:**

The CNA events of the SWI/SNF components are a key process linked to oncogenesis, immune infiltration and therapeutic responsiveness across human cancers.

**Supplementary Information:**

The online version contains supplementary material available at 10.1186/s41065-021-00203-y.

## Introduction

Combinations of somatic genetic alterations, including single base substitutions, translocations, and copy number alterations (CNAs) in essential genes and pathways can lead to the development of cancer [1, 2]. Somatic CNAs, which affect a greater fraction of the cancer genome than other types of somatic changes, play essential roles in promoting cancer progressions [3, 4]. Hence, comprehensive characterization of SCNAs in known cancer-related genes may shed new lights on the cellular defects in cancer and suggest potential treatment strategies.

Previous studies have revealed the high prevalence of alterations in genes encoding chromatin regulatory proteins, implicating them as key regulators in the pathogenesis of human cancers [5]. Among these genetic changes, alterations in genes encoding subunits of SWI/SNF (SWItch/Sucrose NonFermentable), one of the ATP-dependent chromatin remodeling complexes, are present in more than 20% of all human cancers [6, 7]. The mammalian SWI/SNF complexes are present in three distinct final-form complexes, including the canonical BRG1/BRM- associated factor (BAF) complexes (cBAF), polybromo- associated BAF (PBAF) and a newly-characterized non-canonical BAF (ncBAF). These BAF complexes share a group of “core” and “accessory” subunits: SMARCB1, SMARCC1, SMARCC2, SMARCE1, SMARCA4, SMARCA2, and so on [8]. At least 9 different SWI/SNF subunits have been identified as being recurrently mutated across a spectrum of human cancers [9]. For example, the mutational patterns of ARID1A and ARID1B, which are found specifically in the cBAF subunit, have been reported in various cancers. As one of the most frequently mutated genes, ARID1A is the most frequent target of mutations in ovarian, gastric and lung cancers [10], while the mutation of ARID1B is rarer than that of ARID1A [11]. Similarly, mutations of PBRM1, which encodes a subunit of the PBAF complex, were commonly observed in renal carcinoma [12]. Interestingly, mutations affecting components of the ncBAF complex seem to be observed less frequently [13]. Although genes encoding the subunits of the SWI/SNF complex have emerged as broadly mutated in human malignancies, the SCNA events of the complex and the associated clinical relevance has not been well-characterized.

Here, we extensively explored the copy number profiles of 29 genes encoding the SWI/SNF complexes across 33 human cancer types from The Cancer Genome Atlas (TCGA) project. Moreover, clinical information, transcriptome profiles and other genomic features from all the cancers were integrated to functionally characterize the implications and underlying molecular mechanisms of SCNA events of the SWI/SNF complex.

## Materials and methods

### Data collection and processing

All the analyses were conducted using R software newer than version 3.4.2. We obtained the gene expression profiles, copy number data and clinical data from published TCGA data [14].

The number of silent mutation, immunologic mutation, the CNV burden scores, loss of heterozygosity (LOH) scores and homologous recombination deficiency (HRD) scores were derived from published research data [14].

To evaluate the functional states of cancer cells, we downloaded the normalized RNASeqV2 data (https://gdc.cancer.gov/about-data/publications/pancanatlas) and calculated 14 manually curated cancer-related signatures [15] (stemness, invasion, metastasis, proliferation, EMT, angiogenesis, apoptosis, cell cycle, differentiation, DNA damage, DNA repair, hypoxia, inflammation and quiescence) using the GSVA package in R. Moreover, based on the normalized RNASeqV2 data, we applied single-sample gene set enrichment analysis (ssGSEA) [16], to quantify immune cell infiltration in the tumor microenvironment.

The gene sets for cytolytic activity (granzyme-A and perforin-1) and the IFN-γ signature were described in a previous study [17, 18]. The immune signatures were measured as the geometric mean of gene expression in log10 value of the normalized RNA profiling data.

### Statistical analysis

Group values were compared by Student’s t test for normally distributed data, and nonparametric tests were performed when the data were not normally distributed. *P* < 0.05 was defined as statistically significant. We conduct Kaplan–Meier survival curves to estimate overall survival (OS). Statistical analyses were performed using R software and SPSS version 22.0.

## Results

### Widespread CNAs of SWI/SNF complex components across human cancers

**Fig. 1:**
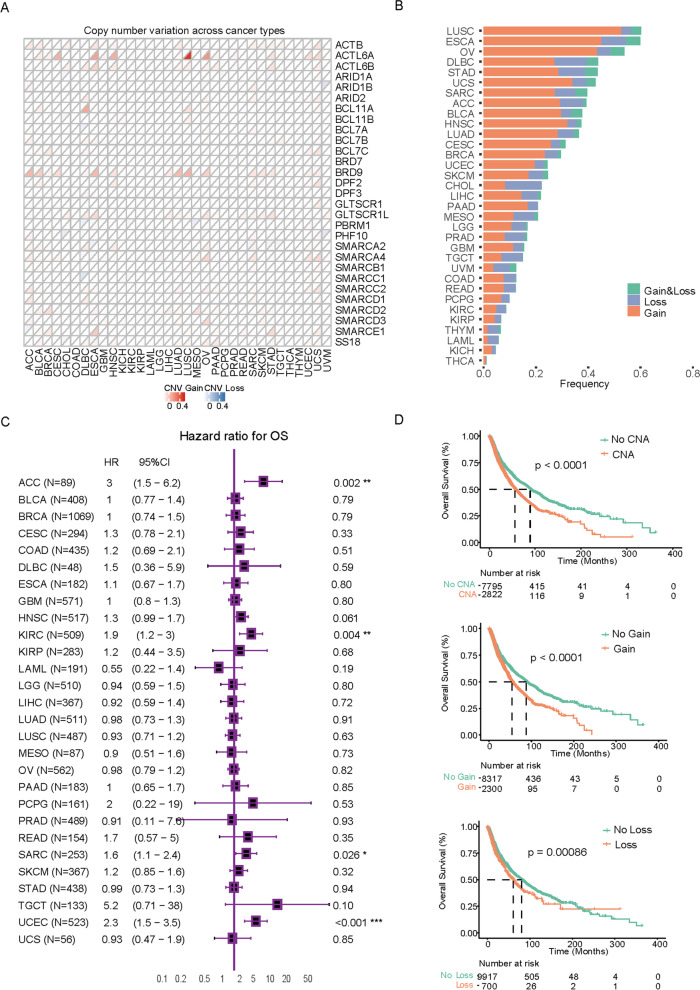
Copy number changes of SWI/SNF complex components across human cancers. **A** CNV profile including amplification (CNV: gain) and deletion (CNV: loss) about each gene in each cancer. **B** Percentages of CNA events including gains (orange) and losses (blue) of SWI/SNF complex in each cancer type. **C** Forest plot showing the hazard ratios (HRs) and 95% confidence interval (CIs) for the associations of CNAs of SWI/SNF complex components. **D** Kaplan-Meier survival analysis comparing OS between patients with or without CNAs of SWI/SNF complex components

We first analyzed the prevalence of copy number variations (CNVs) in genes encoding the subunits of the SWI/SNF complex, such as the cBAF, PBAF, ncBAF, and shared subunits. We identified that the CNA events of the SWI/SNF pathway existed in 26% of all queried cancers, ranging from 0.4 to 6% (Fig. 1A and Figure S1). The most frequently amplified genes were ACTL6A, BRD9, BICRAL and SMARCD2, which belong to the ncBAF or shared subunits. On the other hand, deep deletions were more commonly observed in genes encoding the cBAF or PBAF component, such as, ARID1B, PHF10, PBRM1 and ARID1A (Figure S1). Lung squamous cell carcinoma (LUSC), esophageal carcinoma (ESCA), ovarian cancer (OV) and lymphoid neoplasm diffuse large B-cell lymphoma (DLBC) exhibited relatively higher CNV events in the SWI/SNF pathway; however, lower frequencies were observed in kidney renal clear cell carcinoma (KIRC), kidney renal papillary cell carcinoma (KIRP), thymoma (THYM), acute myeloid leukemia (LAML), kidney chromophobe (KICH), and thyroid carcinoma (THCA) (Fig. 1B).

**Fig. 2:**
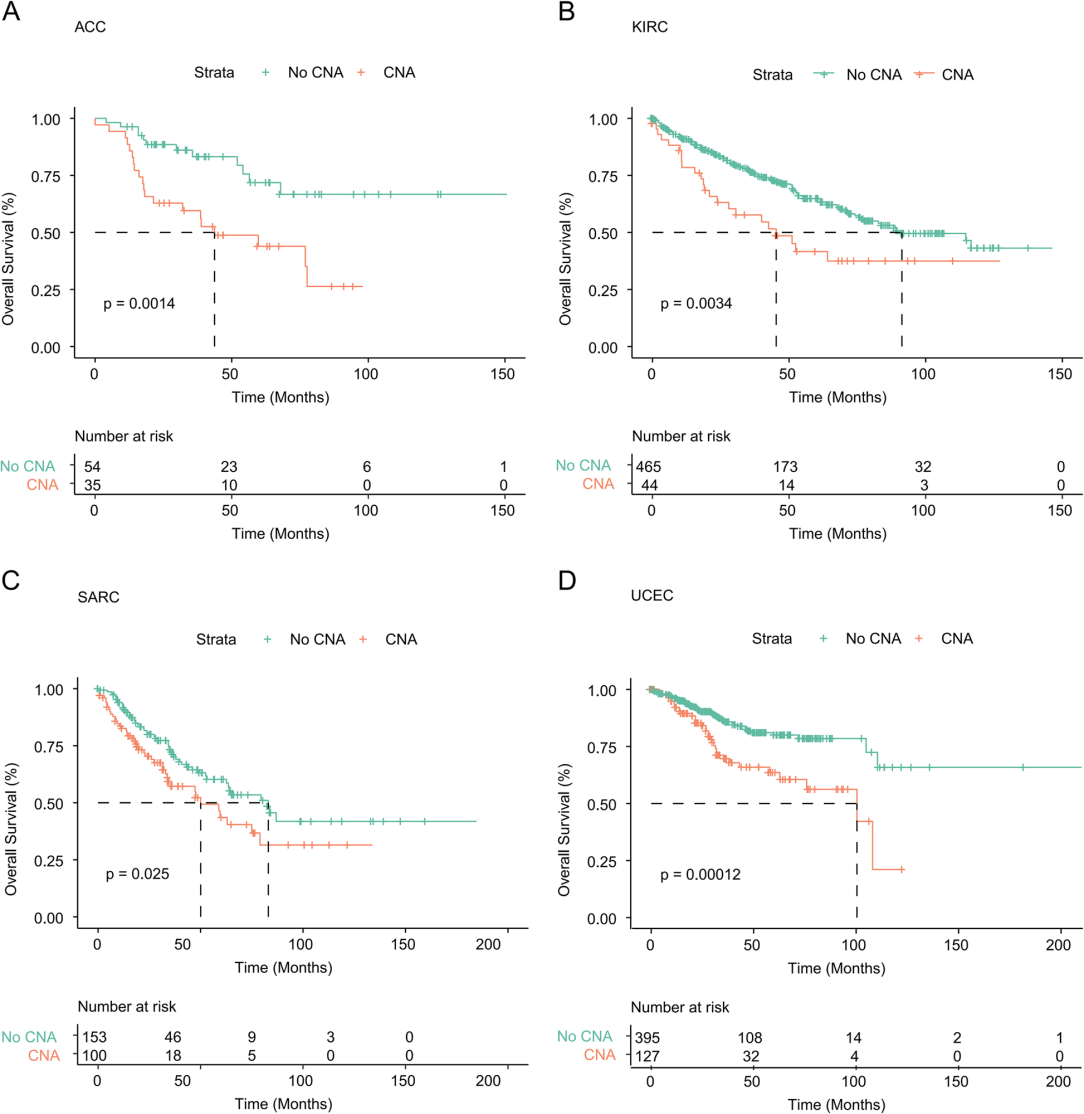
Clinical relevance of CNV gain and/or loss of SWI/SNF complex components in different cancers. **A-D** Kaplan-Meier survival analysis comparing OS between patients with or without CNAs of SWI/SNF complex components in ACC (**A**), KIRC (**B**), SARC (**C**), and UCEC (**D**)

Next, we comprehensively investigated the clinical relevance of the CNA events of the SWI/SNF complex across the 33 cancer types (Fig. 1C and D). Notably, the CNV alterations were unfavorable prognostic factors for multiple cancers, including adrenocortical carcinoma (ACC), sarcoma (SARC), uterine corpus endometrial carcinoma (UCEC) and KIRC (Figs. 1C and 2; log-rank *P* < 0.05 for all comparisons). Next, we explored the clinical relevance of CNV gain and/or loss in the combined TCGA data sets. As revealed in Fig. 1D, cancers with CNV gains, loss or both displayed a decreased median overall survival (OS), indicating that CNV alterations of SWI/SNF complex components may be key regulators implicated in cancer development and progression.

### Distinct genomic characteristics associated with CNAs occurring in the SWI/SNF pathway

Because SWI/SNF CNAs showed a negative impact on clinical outcomes in several cancer types, we further evaluated the distinct genomic profiles associated with these alterations. Previously, some cancers with SWI/SNF gene mutations may exhibit higher rates of microsatellite instability-high (MSI-H) or tumor mutation burden (TMB)-high [19, 20]. However, how SWI/SNF CNAs correlate with these genomic features remains elusive.

**Fig. 3:**
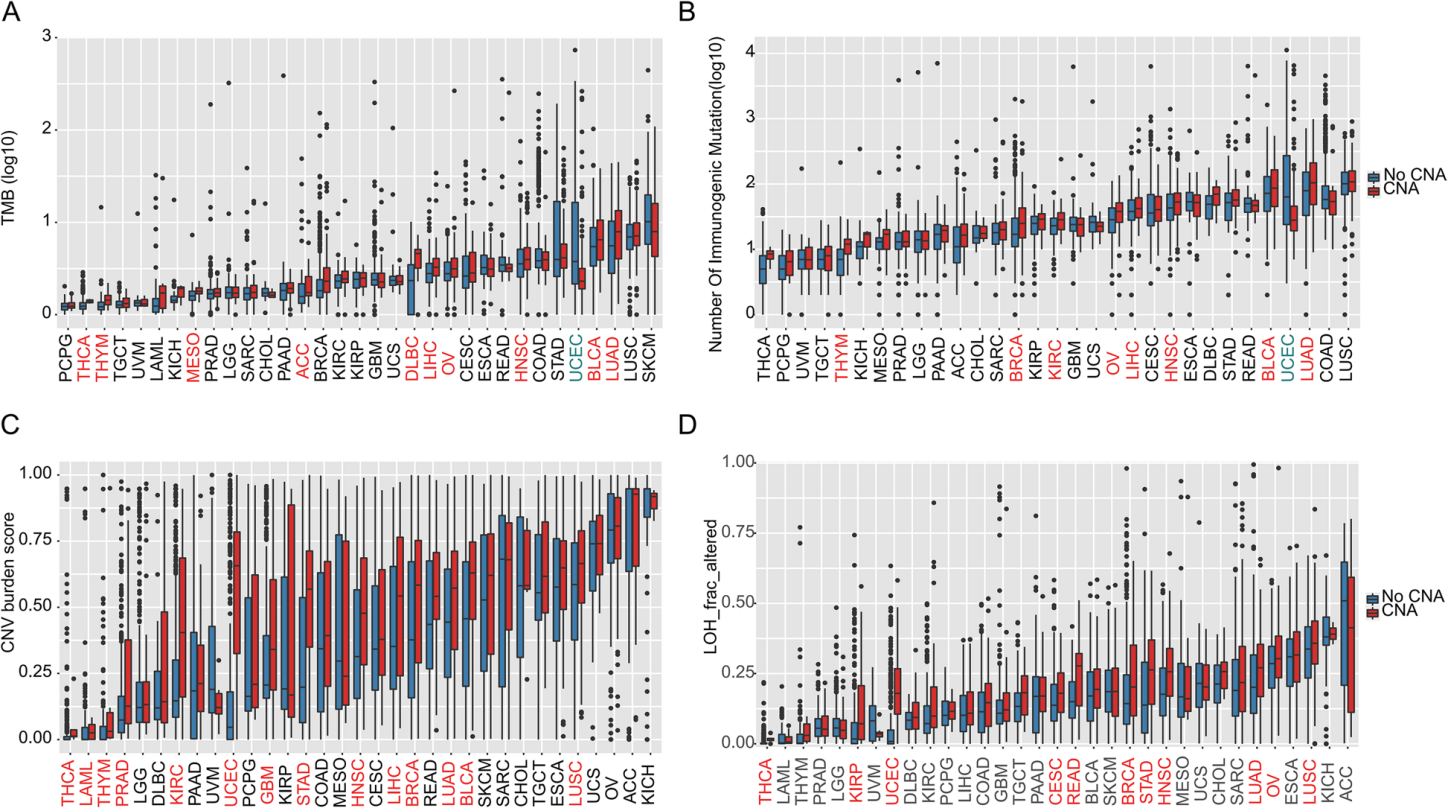
Association of SWI/SNF CNAs with genomic features in different cancers. **A-D** Boxplot comparing TMB (**A**), number of immunologic mutations (**B**), CNV burden score (**C**), and fraction of the genome altered by LOH (LOH_frac_altered) (**D**) between patients with or without CNAs of SWI/SNF complex. (red: upregulated in samples with CNAs; green: downregulated in samples with CNAs, *P* < 0.05, Mann-Whitney U test)

Here, we showed that several tumors (including THYM, OV, LIHC, HNSC, BLCA and LUAD) with SWI/SNF CNV alterations demonstrated significantly higher levels of TMB (Fig. 3A) and tumor neoantigen burden (TNB, Fig. 3B) than the subtypes without CNAs (*P* < 0.05). Interestingly, opposite observations were found in UCEC, in which lower TMB and TNB were identified in the CNA subgroup (Fig. 3A and B). Next, we found that CNV alterations in the SWI/SNF complex were usually correlated with an elevated CNV burden and a larger fraction of loss of heterozygosity (LOH) (Fig. 3C and D), indicating severer DNA damage in mutant tumors. Interestingly, tumors with SWI/SNF CNAs tend to show lower incidence of MSI-H than the control group (Fig. 3E), suggesting a different regulatory role for CNA and SNV in the SWI/SNF pathway.

### SWI/SNF CNAs correlate with homologous recombination deficiency and clinical outcomes of platinum-treated ovarian cancer

**Fig. 4:**
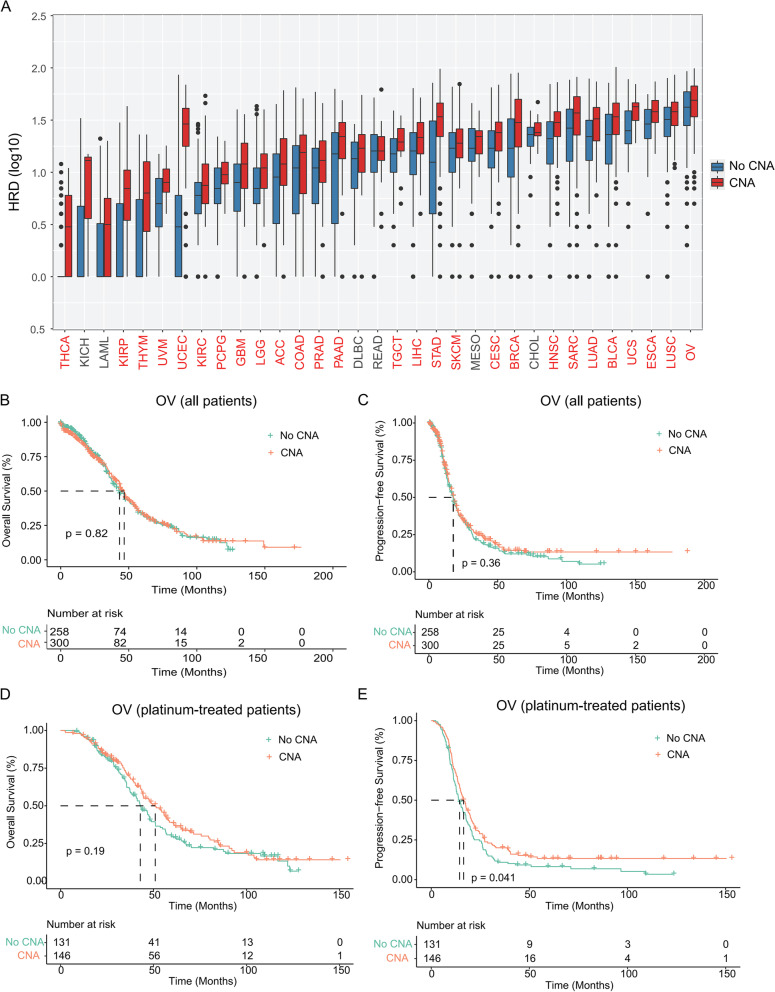
The SWI/SNF CNAs were associated with HRD and improved clinical outcomes of platinum-treated ovarian cancer. **A** Boxplot comparing HRD score between patients with or without CNAs of SWI/SNF complex components (red: upregulated in samples with CNAs; green: downregulated in samples with CNAs, *P* < 0.05, Mann-Whitney U test). **B-C** Kaplan-Meier survival analysis comparing OS (**B**) and PFS (**C**) between patients with or without CNAs of SWI/SNF complex components in all patients of the OV data set. **D-E** Kaplan-Meier survival analysis comparing OS (**D**) and PFS (**E**) between patients with or without CNAs of SWI/SNF complex components in platinum-treated patients from the OV data set

Homologous recombination (HR) and non-homologous end joining (NHEJ) are two major mechanisms for double-strand DNA break (DSB) repair [21]. DSB repair occurs in the context of chromatin; therefore, chromatin regulators such as the SWI/SNF complex may play important roles in the repair process, particularly by HR [22, 23]. To gain further evidence, we first examined the distribution of homologous recombination deficiency (HRD) scores between the mutant (SWI/SNF CNAs) and control subgroups across human cancers. Notably, a large proportion of cancers demonstrated significantly higher HRD scores in the mutant subgroups versus the control (Fig. 4A). This finding indicated that tumors with CNAs occurring in the SWI/SNF pathway might have DNA repair defects and are sensitive to DNA-damaging therapeutics [24, 25].

To test our hypothesis, we performed Kaplan-Meier (KM) survival analysis of ovarian cancer (OV) patients with and without platinum-based chemotherapy. As expected, we observed no significant difference in OS or progression free survival (PFS) between mutant (SWI/SNF CNAs) and control subgroups (Fig. 4B and C). However, ovarian cancers with SWI/SNF CNAs tend to show improved OS (*P* = 0.19) and PFS (*P* = 0.041) in patients treated with platinum-based chemotherapy (Fig. 4D and E), suggesting a novel prognostic role for SWI/SNF CNAs in platinum-treated ovarian cancer.

### Dysregulated oncogenic pathways associated with SWI/SNF CNAs

**Fig. 5:**
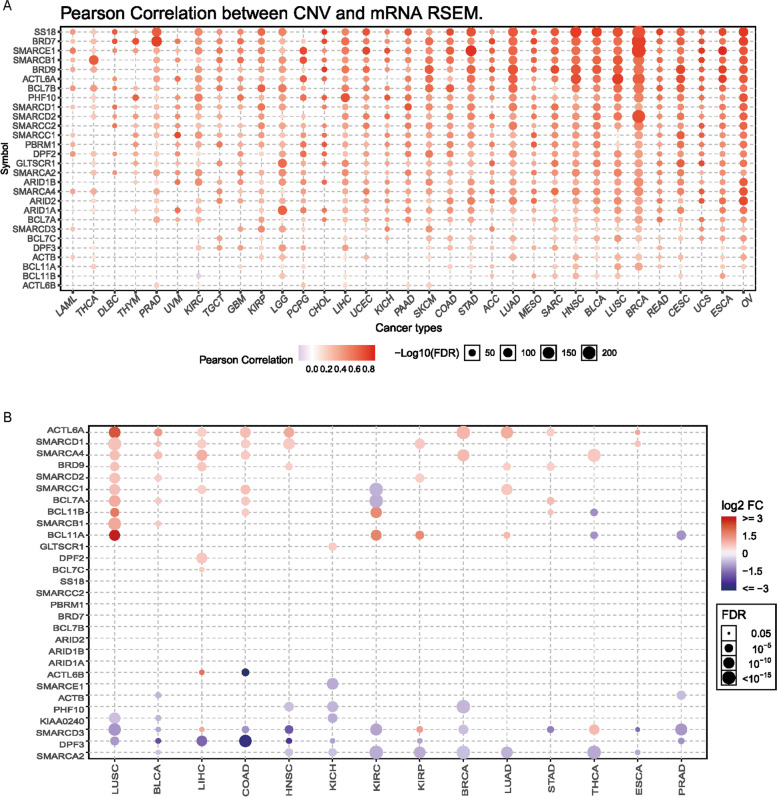
Analysis of gene expression of the SWI/SNF subunits by using the online GSCALite resource. **A** The association between paired mRNA expression and CNV percent samples, based on Person’s product moment correlation coefficient, and follows a t distribution. *P*-value was adjusted by FDR. **B** Differential gene expression of the SWI/SNF subunits in paired tumor and normal samples of the TCGA datasets (only 14 cancer types have over ten paired tumor and normal samples were included). The fold change is mean (Tumor) / mean (Normal), *p*-value was used t-test and *p*-value was adjusted by FDR. The genes with fold change (FC > 2) and significance (FDR > 0.05) were retained for the figure production. All analyses were performed by using the GSCALite resource (http://bioinfo.life.hust.edu.cn/web/GSCALite/)

To further clarify the molecular mechanisms by which SWI/SNF CNAs are implicated in cancer, we investigated the correlation between SCNAs and the enrichment of central pathways in cancers by integrative analysis of the transcriptomic data from the TCGA. As expected, we observed a strong association between paired mRNA expression and CNVs present in tumor samples (Fig. 5A), indicating that the functional impact of SWI/SNF CNAs in tumor progression may result from dysregulated mRNA expression in these genes. Accordingly, the upregulation of mRNAs (in 14 cancer types with available paired tumor and normal samples) encoding SWI/SNF components was found in LUSC, BLCA and LUAD (Fig. 5B), in which higher frequencies of CNV alterations were identified (Fig. 1B). Specifically, ACTL6A, SMARCD1, SMARCA4 and BRD9 are among the top upregulated genes in cancers compared with the paired control samples (Fig. 5B).

**Fig. 6:**
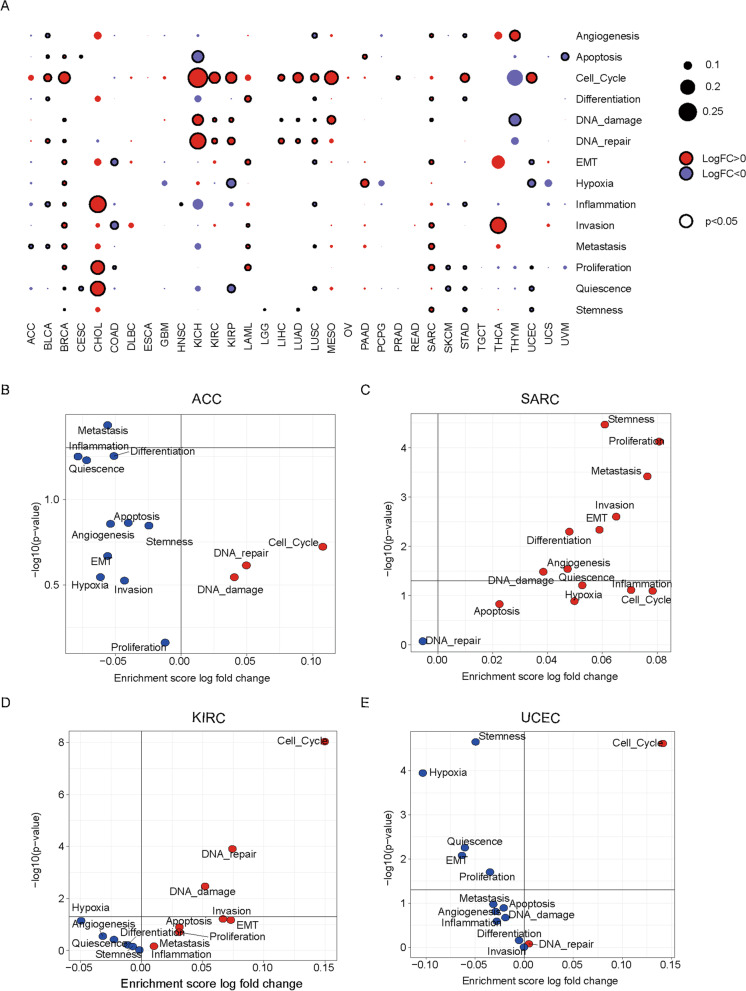
Correlation between SWI/SNF CNAs and cancer-related functional states. **A** The fold change (FC) and significance (p value) of enrichment scores of each signature (14 manually curated cancer-related functional states) was compared between samples with and without CNAs in all cancer types by using the limma R package. **B** Dysregulated cancer-related functional signatures in ACC, SARC, KIRC and UCEC

Next, we explored the distinct functional states of cancer cells between samples with and without CNV alterations in the SWI/SNF complex, such as the cell cycle, proliferation, apoptosis, invasion, stemness, DNA damage, hypoxia and so on. These functional gene sets were obtained from CancerSEA, a cancer single-cell state atlas online database [15]. Interestingly, we observed that several cancer types revealed significantly altered oncogenic pathways (Fig. 6A). For example, enrichment of cell cycle, DNA repair, and DNA damage were commonly observed in multiple cancers, such as, KICH, KIRP, KIRC, LUAD and LUSC (Fig. 6A and Figure S2). These observations agreed with our findings of a significantly higher HRD score in the mutant tumors in most cancer types (Fig. 5A).

Since CNV alterations were unfavorable prognostic factors for 4 cancer types (Fig. 1C), we then asked whether certain common oncogenic features exist among them. Unexpectedly, we found significant heterogeneous mechanisms underlying the CNV alterations of SWI/SNF. In ACC, no significantly upregulated oncogenic pathways were identified; an increased cell cycle signature was observed in KIRC and UCEC, while more than half of the 14 oncogenic pathways were significantly enriched in SARC, such as stemness, proliferation, metastasis, and invasion (Fig. 6B). These observations indicate that CNV alterations in genes encoding the SWI/SNF component may regulate tumor progression via different mechanisms.

### Immunophenotypes associated with SWI/SNF CNAs

Solid tumors and their infiltrating immune cells can interact in a dynamic equilibrium, which, in turn, shape the progression process of cancers [26]. Therefore, we comprehensively analyzed the immune infiltrating pattern and immunophenotypes associated with CNV alterations in the SWI/SNF pathway. First, we observed no significant changes in the stroma fraction, cytolytic activity, and PD-1 expression between CNA versus control subgroups in all inquired tumor samples; however, IFNG, GZMA and PD-L1 expression were elevated in mutant (CNA) cancers (Figure S3). The leukocyte fraction (LF) has been shown to vary across distinct immune subtypes, and tumors with the top LF tend to be most responsive to immune checkpoint blockades (ICBs) [14]. Here we showed that higher levels of LF only correlated with SWI/SNF CNAs in a few tumor types, including USC, UCEC, COAD and BRCA (Fig. 6A).

**Fig. 7:**
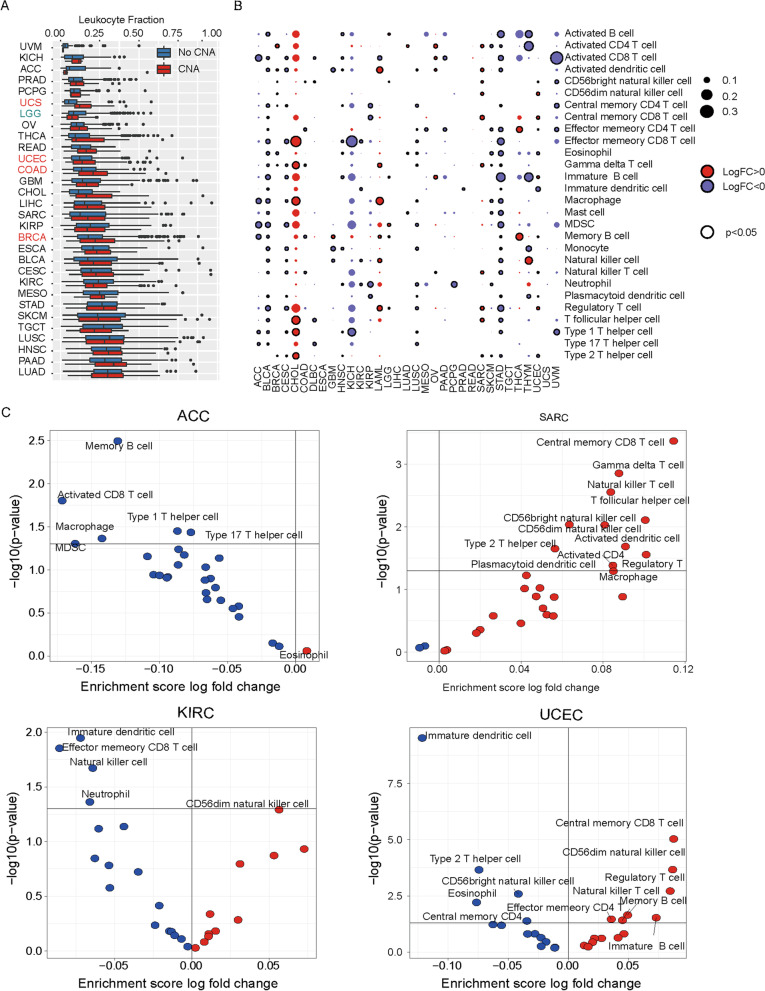
Correlation between SWI/SNF CNAs and immune cell infiltration. **A** Boxplot comparing leukocyte fraction between patients with or without CNAs of SWI/SNF complex components (red: upregulated in samples with CNAs; green: downregulated in samples with CNAs, *P* < 0.05, Mann-Whitney U test). **B** The fold change (FC) and significance (*p* value) of enrichment scores of immune cells was compared between samples with and without CNAs in all cancer types by using the limma R package. **C** Dysregulated immune cell infiltration in ACC, SARC, KIRC and UCEC

Next, we explored how SWI/SNF CNAs reshape the composition of tumor immune infiltrations across all cancers. Intriguingly, we observed two distinct patterns that revealed opposite associations of SWI/SNF CNAs with tumor-infiltrating lymphocytes (TILs). Specifically, significant enrichment of TILs was observed in CHOL, LAML and SARC when CNAs occur in the SWI/SNF complex, suggesting an “immune-inflamed” phenotype (Fig. 7B and Figure S4). By contrast, a negative correlation was found in KICH, KIRC, STAD, ACC and UVM, and so on. No significant associations were identified in LIHC and PRAD. Interestingly, although SWI/SNF CNAs correlated with unfavorable clinical outcomes in the four tumor types (Fig. 2), they revealed different immunophenotypes in the SWI/SNF mutant subgroups, supporting different mechanisms for the SWI/SNF CNAs in regulating both tumor cells and immune infiltration.

## Discussion

Recently, the genomic alterations as mutations and translocations involving the SWI/SNF complex have been linked to several human cancers [27]. Importantly, several studies have revealed a correlation between the SWI/SNF mutation and antitumor immunity and that gene mutations of this complex may have a higher likelihood of therapeutic response to immune checkpoint inhibitors [19, 20]. However, the somatic copy number gains and losses of the complex and associated clinical relevance have not been systematically investigated. In the present study, we systematically characterized the SCNA events of the 29 genes encoding the SWI/SNF complexes across 33 human cancer types from TCGA. We observed that SWI/SNF CNAs are associated with the enrichment of gene sets belonging to the cell cycle, DNA damage and repair, and dysregulated immune infiltration as well, which may collectively result in inferior clinical outcomes in several cancer types. Additionally, substantial discrepancies have been found across different tumor types.

In the present study, we observed that copy number changes, particularly CNV gains, are widespread genomic events across different human cancer types (Fig. 1 and Figure S1). Previously, focal amplifications of BRD9 and ACTL6A have been identified and their overexpression may correlate with unfavorable clinical outcomes in a set of cancers. However, ARID1A, ARID1B and PBRM1 are top-ranked genes with copy number losses (Fig. 1 and Figure S1). Interestingly, loss of either ARID1A or PBRM1 expression has been associated with abnormal processes of DNA damage repair (DDR) [13, 28]. As a result, loss of ARID1A, ARID1B and PBRM1 are usually indicators of poor clinical outcomes [29–31]. Collectively, these observations indicate that SCNA events (either gains and losses) of the SWI/SNF complex might be key aspects of aggressive tumor behavior and lead to worse clinical outcome (Fig. 1D).

To understand which aspects of cancer biology might be influenced by SCNAs in the SWI/SNF complex, we comprehensively analyzed the genomic features and oncogenic pathways across all cancer types. We first found a positive correlation between SWI/SNF CNAs and several genetic features, including the TMB, TNB, CNV burden score and LOH score, in a large proportion of cancer types (Fig. 3), suggesting a higher level of aneuploidy and genome instability. Interestingly, a negative correlation between mutations and SWI/SNF CNAs was only observed in UCEC. Different SWI/SNF family members have been shown to play distinct roles in DNA damage and repair processes, ranging from modifying chromatin structure to directly recruiting repair factors [32, 33]. Therefore, we next explored how HRD might be affected by SWI/SNF CNAs. Notably, a significant positive correlation between the HRD score and SCNAs in the SWI/SNF complex was identified in 27/33 cancer types. More importantly, we presented a novel finding that SWI/SNF CNAs may function as a prognostic marker for platinum-based chemotherapy in ovarian cancer (Fig. 4D-E), an observation that has not been reported previously. Our data suggest that tumors harboring the SWI/SNF CNAs may have DNA repair defects and may be sensitive to DNA-damaging therapeutics.

Next, we investigated how SCNA events in the SWI/SNF complex may be related to oncogenic pathways [15] and immune infiltration [14]. Tumors are complex ecosystems comprising cancer cells, endothelial cells, infiltrating immune cells and extracellular matrix. Cross-talk among these components shapes tumor development [34]. Generally, we found that tumors with SWI/SNF SCNAs showed enrichment of the cell cycle, DNA damage and repair (Fig. 6). By contrast, a negative correlation was found in immune cell infiltrates in a large proportion of cancer types (Fig. 7). Our data indicated that the SWI/SNF CNAs might be a driver event for tumorigenesis. Since SWI/SNF CNAs were associated with worse clinical outcomes in four cancer types (Fig. 2), we then asked whether the SWI/SNF alterations act via the same mechanisms. Interestingly, oncogenic markers and immune signatures revealed distinct patterns in the four cancer types. In ACC, although SWI/SNF CNAs did not increase the oncogenic signatures such as the cell cycle or DNA damage and repair, they were associated with decreased immune infiltration including that of the activated CD8 T-cells. Previously, ACC was classified as a lymphocyte-depleted subtype that displays composite signatures reflecting an immunosuppressed tumor microenvironment (TME) [14]. Here we showed that the SWI/SNF CNAs might result in reduced immune infiltration and confer a worse prognosis (Figs. 2A and 7C). However, in SARC, SWI/SNF CNAs showed a strong positive correlation with both oncogenic pathways and infiltrating immune cells (Figs. 6B and 7C), indicating a broken balance between immune control and tumor growth. Notably, in KIRC, we observed a strong enrichment in oncogenic signatures and impaired immune infiltration in the mutant subgroup, a finding that may collectively explain why SWI/SNF CNAs are risk prognostic factors. Although SCNA events of the SWI/SNF complex have indicated a worse OS in UCEC, unexpectedly, we found that several oncogenic signatures, including stemness, hypoxia, EMT and proliferation, were significantly decreased in the SWI/SNF CNA subgroup. Therefore, it would be of great interest to clarify the underlying and heterogeneous mechanisms across these cancer types.

## Conclusions

In conclusion, we delineated the prevalent copy number alterations of the SWI/SNF complex components across various human cancer types. Notably, the SCNA events in the SWI/SNF complex are correlated with dysregulated oncogenic pathways and immune infiltration, and may lead to unfavorable clinical outcomes for multiple cancers. Strikingly, we also presented novel evidence that tumors with CNAs occurring in the SWI/SNF pathway may have DNA repair defects and may be sensitive to DNA-damaging therapeutics.

## Supplementary Information

**Additional file 1: Figure S1.** Landscape of SWI/SNF CNAs in 33 TCGA cancer types by using the cBioportal database.

**Additional file 2: Figure S2.** Correlation between SWI/SNF CNAs and cancer-related functional states in different cancer types.

**Additional file 3: Figure S3.** Correlation between SWI/SNF CNAs and immune signatures in all TCGA datasets. *P* < 0.05, Mann-Whitney U test.

**Additional file 4: Figure S4.** Correlation between SWI/SNF CNAs and immune cell infiltration in different cancer types.

## Data Availability

The datasets used and analyzed during the current study are available from the corresponding author on reasonable request.

## Abbreviations

CNAs: Copy number alterations
SWI/SNF: SWItch/Sucrose NonFermentable
BAF: BRG1/BRM- associated factor
cBAF: BRG1/BRM- associated factor complexes
PBAF: Polybromo- associated BRG1/BRM- associated factor
TCGA: The Cancer Genome Atlas
LOH: Loss of heterozygosity
HRD: Homologous recombination deficiency
ssGSEA: Single-sample gene set enrichment analysis
CNV: Copy number variation
LUSC: Lung squamous cell carcinoma
ESCA: Esophageal carcinoma
OV: Ovarian cancer
DLBC: Lymphoid neoplasm diffuse large B-cell lymphoma
KIRC: Kidney renal clear cell carcinoma
KIRP: Kidney renal papillary cell carcinoma
THYM: Thymoma
LAML: Acute myeloid leukemia
KICH: Kidney chromophobe
THCA: Thyroid carcinoma
ACC: Adrenocortical carcinoma
SARC: Sarcoma
UCEC: Uterine corpus endometrial carcinoma
OS: Overall survival
MSI: Microsatellite instability
TMB: Tumor mutation burden
TNB: Tumor neoantigen burden
HR: Homologous recombination
NHEJ: Non-homologous end joining
DSB: Double-strand DNA break
KM: Kaplan-Meier
PFS: Progression free survival
LF: Leukocyte fraction
TIL: Tumor-infiltrating lymphocyte
DDR: DNA damage repair
