# Supplementary figures and images for: Comprehensive characterization and clinical relevance of the SWI/SNF copy number aberrations across human cancers
Source: Hereditas. 2021 Oct 1;158:38. doi: 10.1186/s41065-021-00203-y (PMC8487138; doi:10.1186/s41065-021-00203-y)

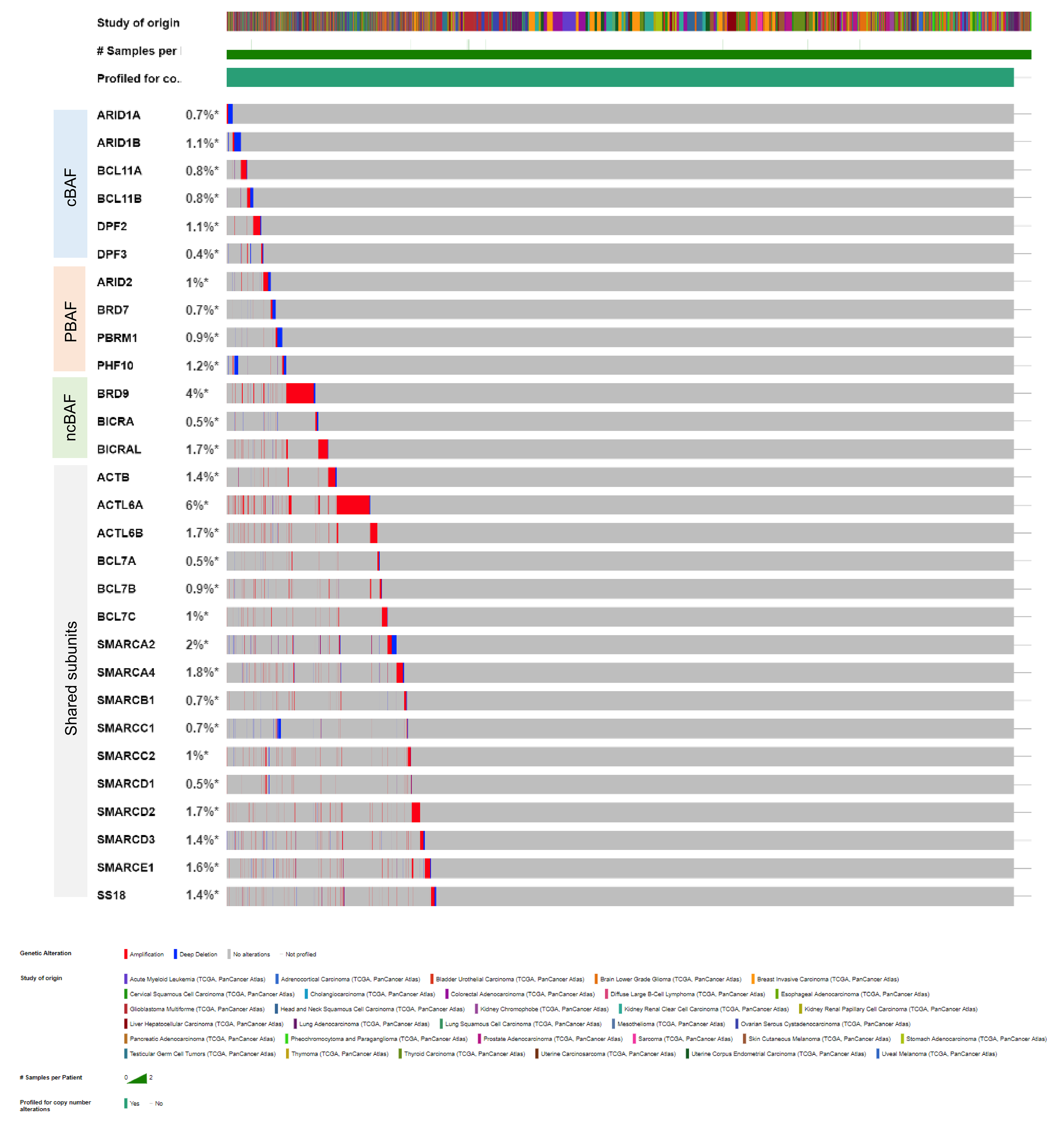

Supplement: Supplementary file 1 — Additional file 1: Figure S1. Landscape of SWI/SNF CNAs in 33 TCGA cancer types by using the cBioportal database. [file 41065_2021_203_MOESM1_ESM.tif]

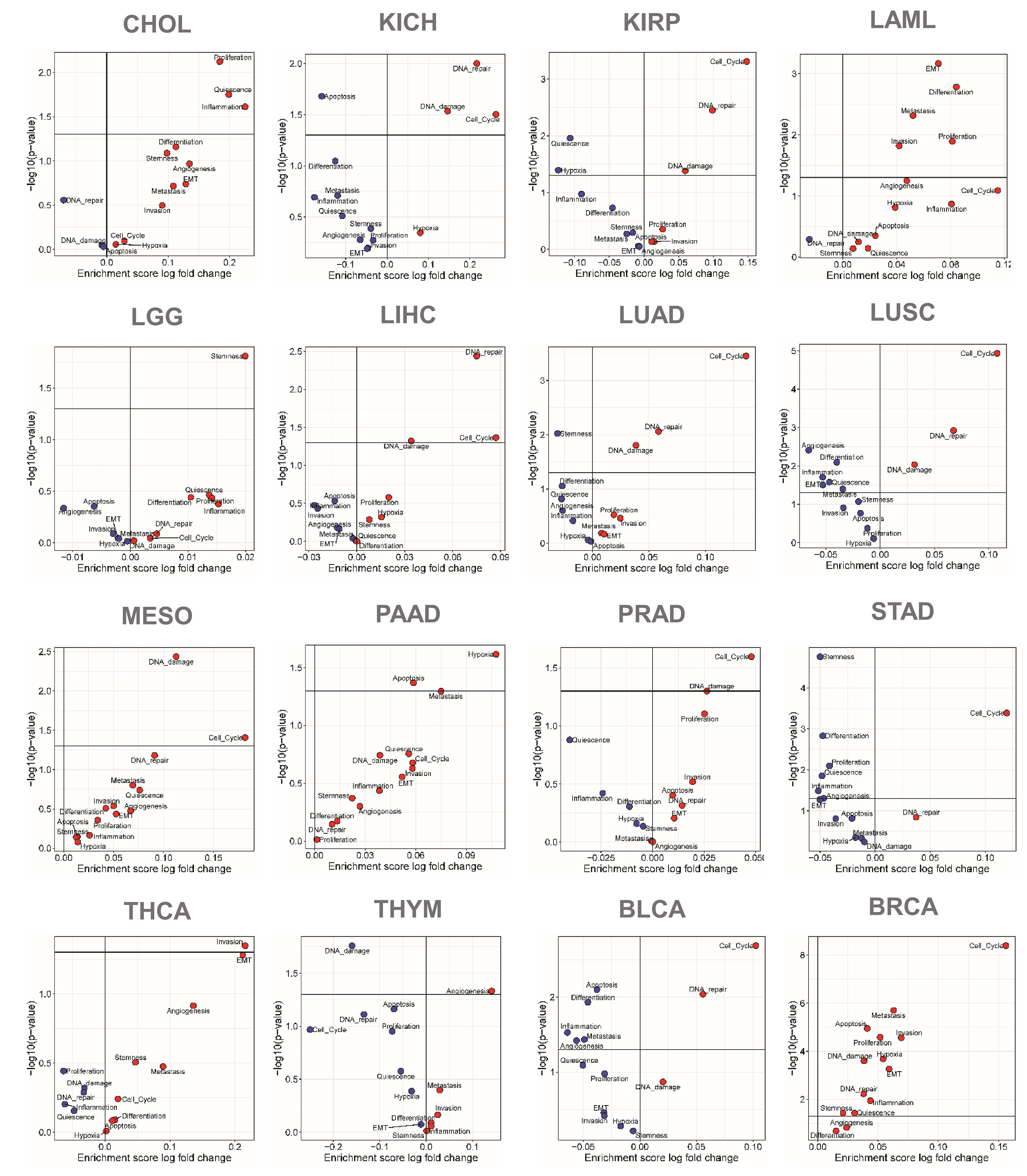

Supplement: Supplementary file 2 — Additional file 2: Figure S2. Correlation between SWI/SNF CNAs and cancer-related functional states in different cancer types. [file 41065_2021_203_MOESM2_ESM.tif]

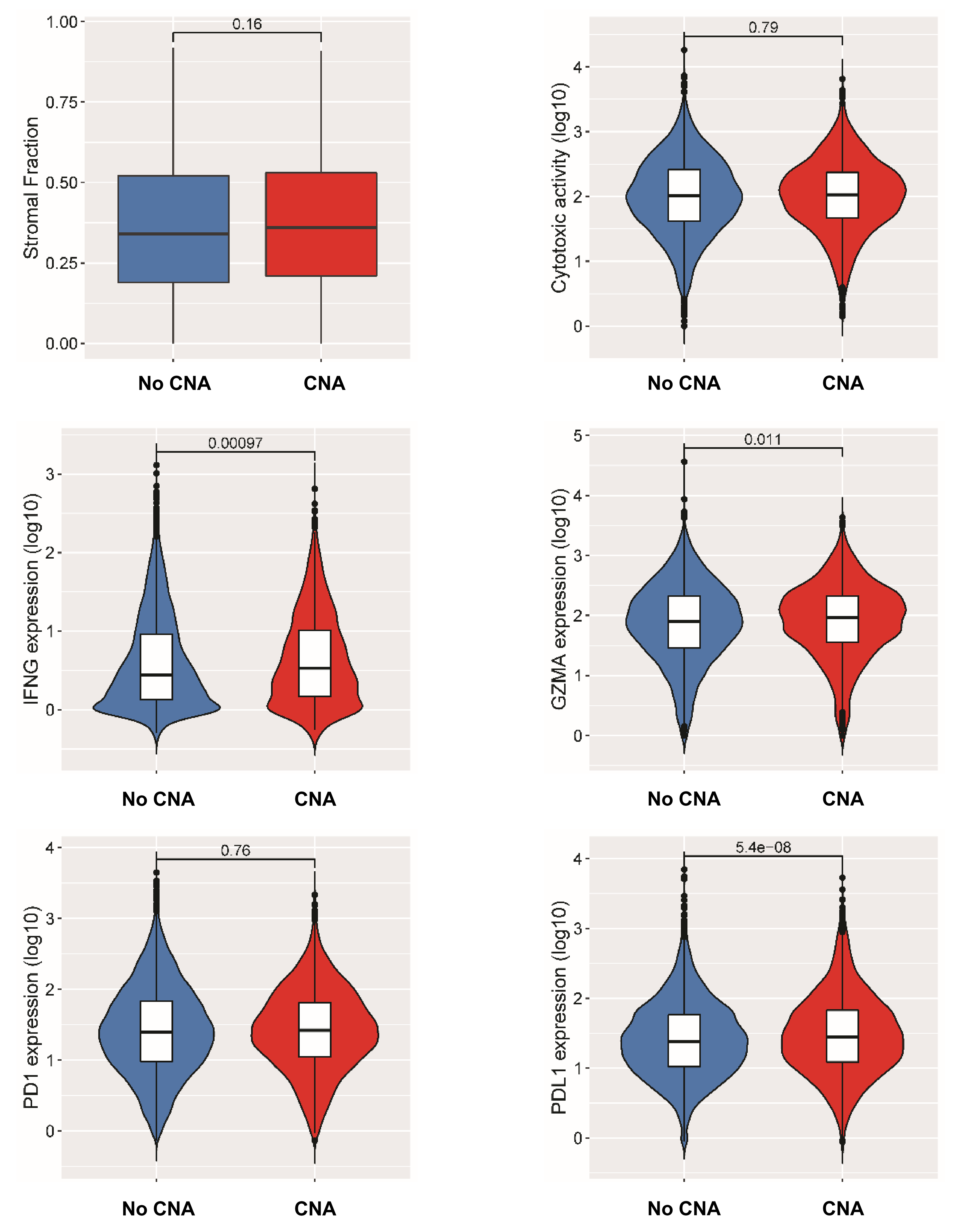

Supplement: Supplementary file 3 — Additional file 3: Figure S3. Correlation between SWI/SNF CNAs and immune signatures in all TCGA datasets. P < 0.05, Mann-Whitney U test. [file 41065_2021_203_MOESM3_ESM.tif]

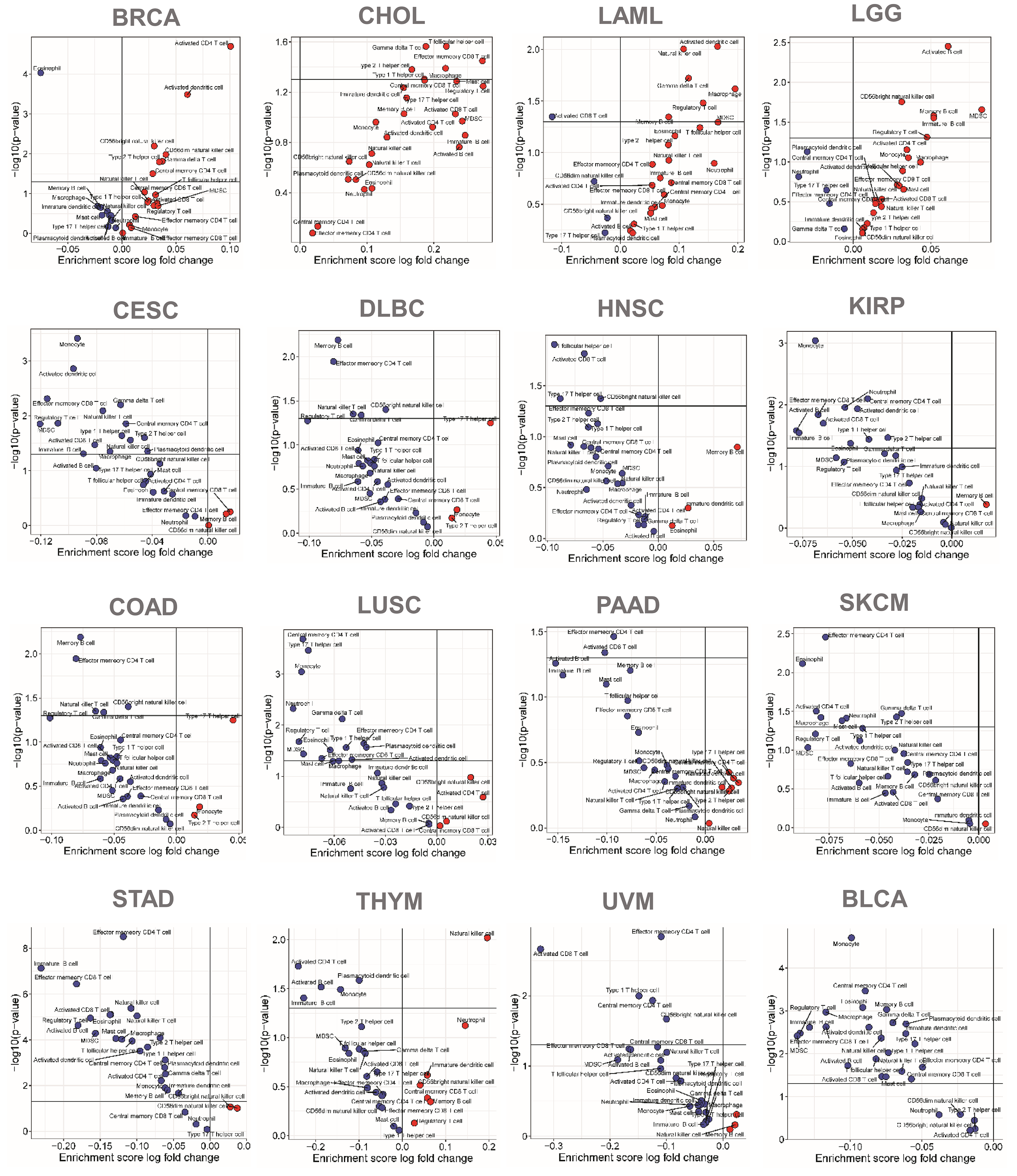

Supplement: Supplementary file 4 — Additional file 4: Figure S4. Correlation between SWI/SNF CNAs and immune cell infiltration in different cancer types. [file 41065_2021_203_MOESM4_ESM.tif]
